# Isolation, characterization, and vascular potential of porcine cells in a three-dimensional decellularized liver matrix model

**DOI:** 10.3389/fbioe.2025.1625999

**Published:** 2025-08-04

**Authors:** Sara Morini, Sandra Melitón Barbancho, Álvaro Blanes Rodríguez, Iris Pla-Palacín, Pilar Sainz-Arnal, Natalia Sánchez-Romero, Maria Victoria Falceto, Olga Mitjana, Antonio Romero, Marcela Del Rio-Nechaevsky, Maria Lourdes Bengochea Martinez, Emma Olmedo Arbizu, Sara Lorente, Angel Lanas, Ana Fernandes-Platzgummer, Pedro M. Baptista

**Affiliations:** ^1^ Department of Bioengineering and iBB – Institute for Bioengineering and Biosciences, Instituto Superior Técnico, Universidade de Lisboa, Lisbon, Portugal; ^2^ Associate Laboratory i4HB - Institute for Health and Bioeconomy at Instituto Superior Técnico, Universidade de Lisboa, Lisbon, Portugal; ^3^ Instituto de Investigación Sanitária de Aragón (IIS Aragón), Zaragoza, Spain; ^4^ Cytes Biotechnologies SL, Barcelona, Spain; ^5^ Facultad de Ciencias de la Salud, Universidad San Jorge, Campus Universitario, Zaragoza, Spain; ^6^ Departamento de Patología Animal, Instituto Agroalimentario de Aragón-IA2 (Universidad de Zaragoza-ITA), Zaragoza, Spain; ^7^ Biomedical Engineering Department, Carlos III University of Madrid, Madrid, Spain; ^8^ CIBERER, Centro de Investigación Biomédica en Red de Enfermedades Raras, Instituto de Salud Carlos III, Madrid, Spain; ^9^ Instituto de Investigación Sanitaria de la Fundación Jiménez Díaz, Madrid, Spain; ^10^ Hospital Clínico Universitario Lozano Blesa, Zaragoza, Spain; ^11^ Centro de Investigación Biomédica en Red en el Área Temática de Enfermedades Hepáticas (CIBERehd), Madrid, Spain; ^12^ Fundación Agencia Aragonesa para la Investigación y el Desarrollo, Zaragoza, Spain

**Keywords:** mesenchymal stromal cells, smooth muscle cells, endothelial cells, decellularized liver ECM, 3D dECM disc vascular model

## Abstract

**Introduction:**

The bioengineering of solid tissues and organs to mitigate the organ donor shortage has become a critical area of research in tissue engineering and regenerative medicine, where establishing a functional vascular network is crucial, particularly for complex organs such as the lung, kidney, and liver. This requires the isolation and characterization of various vascular cell types. In this quest, pigs have emerged as the preferred experimental animal model in this field, highlighting the importance of procuring and characterizing porcine vascular cells to create organs with functional vasculature for transplant. However, species-unique differences present challenges. Although some of the processes for isolating, expanding, and characterizing porcine vascular cells have been published, these are less established than those for human cells, requiring in our view and experience, additional research. Furthermore, no reliable and comprehensive models currently exist for testing vascular cell interactions in co-culture *in vitro*.

**Methods:**

In this study, we developed effective methods to isolate and further characterize distinct porcine vascular cell types from various sources. We also introduced a straightforward and practical three-dimensional model for testing vascular cell co-culture, organization and function* in vitro*.

**Results and discussion:**

This proof-of-concept study demonstrates the potential of our co-culture strategy, employing a decellularized liver extracellular matrix disc scaffold microenvironment to assess cell interactions and vascular potential on a small scale* in vitro*.

## 1 Introduction

Organ transplantation is the sole definitive treatment for end-stage organ disease, yet its success is hampered by a severe shortage of organ donors (Meng et al., 2017). To address this, bioengineered organs produced through organ decellularization and recellularization offer a promising solution (Uygun et al., 2010; Baptista et al., 2011; Yagi et al., 2013). These bioengineered organs can be further matured with various cell types to enhance functionality for transplantation (Caralt et al., 2014).

One critical challenge in the development of bioengineered organs is identifying optimal cell sources that not only minimize or prevent immune rejection in the recipient but also possess the capacity for large-scale *in vitro* expansion to meet the structural and functional demands of fully developed organs. Given these stringent requirements, most studies employing animal models rely on pigs because of their remarkable anatomical similarity to human organs (Welman et al., 2015; Shaheen et al., 2019; Anderson et al., 2021; Citro et al., 2023).

Moreover, for successful organ function, it is fundamental that all components are effectively bioengineered since the overall function of an organ depends on the integration of its constituents, such as epithelia, mesoderm, parenchyma, and vasculature (Jain et al., 2005; Badylak et al., 2011; Pellegata et al., 2018). In this context, the vasculature plays a crucial role, serving as the primary communication link between the organ and the body and preventing thrombogenesis (Ribitsch et al., 2020). It is also vital to guarantee the delivery of oxygen and nutrients to the cells and remove cellular waste products, a critical aspect in tissue and organ bioengineering (Jain et al., 2005).

Creating long-term functional blood vessels within three-dimensional (3D) structures mimicking native tissues is currently the major challenge in tissue-engineered constructs. Blood vessels involve intricate interactions between distinct cell types, including homotypic and heterotypic communications. Moreover, these cells secrete growth factors and cytokines, synthesize and remodel the surrounding extracellular matrix (ECM), and play vital roles in angiogenesis. Numerous efforts have been undertaken in the last decades to study these processes to establish models that target angiogenesis (Goodwin, 2007; Ucuzian and Greisler, 2007). However, often used models like the “tube formation assay” on Matrigel™, fall short of capturing the *in vivo* complexity and relevance (Arnaoutova et al., 2009; Simons et al., 2015; Nowak-Sliwinska et al., 2018). Additionally, other cell types like smooth muscle cells (SMC) and fibroblasts form networks due to cellular traction forces, which do not accurately represent angiogenesis (Arnaoutova et al., 2009). Moreover, other decellularized liver ECM (dECM) studies showed the potential of combining native ECM and endothelial cells (EC) to induce cellular network formation (Mazza et al., 2015). However, the lack of other vascular cell types prevented the formation of more complex structures and interactions. Consequently, more sophisticated and comprehensive 3D models are required to faithfully recreate the three-dimensional angiogenic environment that enables the formation of a multicomponent microvascular network.

Thus, to replicate native vascular physiology more faithfully, we centered this work on a three-dimensional extracellular-matrix (ECM) microenvironment and examined how it modulates the crosstalk among porcine endothelial cells, smooth-muscle cells and mesenchymal stromal cells. We first refined and streamlined existing protocols for isolating, expanding and maintaining each cell type, then subjected the resulting populations to an expanded phenotypic and functional characterization. Building on these optimized cells, we created a proof-of-concept 3D model—based on a liver-derived decellularized ECM scaffold—in which the three lineages were co-cultured, allowing us to evaluate their recellularization potential, mutual interactions and responsiveness to additional cues such as mechanical loading or soluble growth factors. By uniting protocol refinement, comprehensive characterization and 3D co-culture testing, this study offers a robust framework for dissecting vascular-cell dynamics and advancing tissue-engineering (TE), regenerative-medicine (RM) and personalized-therapy applications.

## 2 Materials and methods

### 2.1 Animal tissues

Bone marrow and aorta tissues were obtained from male and female domestic Landrace piglets (weighting 5–7 kg). Umbilical cords were obtained from pig fetuses (72–80 days after insemination) after a planned cesarean section was performed under hospital conditions at the Veterinary School of the University of Zaragoza, Spain. All animals were housed at the Experimental Surgery Department under approved conditions and the tissues were harvested after intraoperative approved euthanasia.

Pigs were sedated with a combination of ketamine (10 mg/kg), medetomidine (0.02 mg/kg), and midazolam (0.1 mg/kg), followed by systematic heparinization. To prevent clot formation during exsanguination, 0.9% sodium chloride solution (Baxter, United States), supplemented with sodium heparin (300 IU/kg, Rovi, Spain), was administered intravenously via the auricular vein using a 24G needle. Anesthesia was subsequently induced through the same auricular vein using an intravenous injection of Propofol (Propovet™, Richmond Vet Pharma, Argentina), up to a maximum dose of 6 mg/kg. Once deep sedation was confirmed, exsanguination was performed under sterile conditions by direct puncture of the external jugular vein, ensuring complete blood drainage prior to tissue collection. The experiments were performed following the European guidelines regarding the protection of animals and were approved by the Animal Experimentation Committee of the Government of Aragón, Spain (ref. PI08/17).

### 2.2 Isolation and cultivation of porcine bone marrow-derived mesenchymal stromal cells (pMSC(M))

Bone marrow-derived mesenchymal cells (pMSC (M)) were harvested under aseptic conditions from the femur of sacrificed and bled piglets (5–7 kg). The whole femur was dissected from the animal and immersed in cold Dulbecco’s Modified Eagle Medium (DMEM) (Gibco, ThermoFisher Scientific, United States) for transportation. In a biosafety cabinet, the bone’s epiphysis was removed at the metaphysis level to expose the bone marrow. An 18-gauge needle attached to a syringe containing DMEM supplemented with 1% (v/v) Penicillin–Streptomycin (100×; Gibco, Thermo Fisher Scientific, United States) was inserted into the femoral medullary cavity. The medium was slowly perfused until the cortical bone blanched, indicating that the marrow had been fully flushed. The extracted bone marrow was diluted with an equal volume of phosphate buffered saline (PBS) (Corning, United States), layered onto the Histopaque solution (1.077 g/mL) (Sigma-Aldrich, Merck, Germany) and centrifuged at 400 × g for 30–40 min with brake turned off. The buffy coat was transferred to a 50 mL centrifuge tube using a sterile Pasteur pipette, diluted with 3 volumes of PBS, resuspended and centrifuged at 400 × g for 10 min. The supernatant was removed, and the cell pellet was resuspended in PBS and centrifuged at 400 × g for 10 min. Then, the supernatant was removed and the cell pellet was resuspended in DMEM + 10% (v/v) Fetal Bovine Serum MSC Qualified (FBS) (Gibco, ThermoFisher Scientific, United States) + 1% (v/v) P/S (DMEM/FBS-MSC) and seeded between 200,000 and 500,000 cells/cm^2^ in 152 cm^2^ Petri dishes (Corning, United States) coated with 0.2% (v/v) gelatin (Sigma-Aldrich, Merck, Germany) at 37°C in a humidified atmosphere of 5% CO_2_. Once confluent (80%), pMSC(M) were either frozen or sub-cultured following cell detachment with 0.05% (v/v) trypsin/0.02% (v/v) EDTA (Gibco, ThermoFisher Scientific, United States) and seeded at 3,000 cells/cm^2^ in 152 cm^2^ Petri dishes coated with 0.2% (v/v) gelatin. Passage 3-5 cells were used in experiments. The photos were captured with an inverted optical microscope (Leica DMI3000B/Nikon Digital Camera Dxm1200F).

### 2.3 Isolation and cultivation of porcine aortic smooth muscle cells (pASMC)

pASMC were isolated using a modified version of the protocol published by Beigi and colleagues from porcine aortas (Beigi et al., 2017). The aortic tube was dissected to eliminate the external elastic lamina and to remove the tunica adventitia. One extremity of the vessel was ligated with a surgical 2–0 silk suture (B. Braun Melsungen, Germany) and checked for leaks with PBS. Next, the aorta was filled with Dispase II protease (Sigma-Aldrich, Merck, Germany) (2.5 U/mL in DMEM) supplemented with 1% (v/v) P/S, and the other end of the tube was ligated. The resulting vessel was incubated for 1 h at 37°C in a humidified atmosphere of 5% CO_2_. Then, the enzyme solution from the aorta was collected using DMEM + 10% (v/v) FBS + 1% (v/v) P/S (DMEM/FBS) and removed to eliminate the EC fraction of the aorta. After this, the tissue was cut open and placed in a Petri dish with the tunica intima facing up and scraped away any remaining EC using a cell scraper (ThermoFisher Scientific, United States). The tunica media layer was cut into 1–2 mm pieces, placed in a 50 mL conical centrifuge tube (Corning, United States), and incubated for 1–1.5 h in 0.2% (w/v) collagenase type I (Sigma-Aldrich, Merck, Germany) in DMEM + 1% (v/v) P/S at 37°C with agitation at 210 rpm on an orbital shaker (Innova 40, New Brunswick Scientific, United States). This collagenase solution was then discarded and replaced with 0.1% (w/v) collagenase type I in DMEM + 1% (v/v) P/S and incubated for 5 h at 37°C with agitation at 230 rpm on an orbital shaker. After 5 h, the cell-enzyme solution was deactivated with an equal volume of DMEM/FBS and filtered through a 100 µm and then a 40 μm cell strainer (Corning, United States) to eliminate large pieces of undigested aortic tissue. The collected cell suspension was centrifuged, and the pellet was resuspended in DMEM/FBS. The obtained pA-SMC were seeded at 6,000–14,000 cells/cm^2^ in 152 cm^2^ Petri dishes coated with 5 μg/mL fibronectin (Sigma-Aldrich, Merck, Germany) in DMEM/FBS at 37°C in a humidified atmosphere of 5% CO_2_. The medium was replaced with fresh medium after 48 h from the initial seeding and was changed every 3 days. Once confluent (90%), cells were either frozen or sub-cultured following cell detachment with 0.05% (v/v) trypsin/0.02% (v/v) EDTA and seeded at 6,000 cells/cm^2^ in 152 cm^2^ Petri dishes coated with 0.2% (v/v) gelatin in DMEM/FBS and let at 37°C in a humidified atmosphere of 5% CO_2_. Passage 3-5 cells were used in experiments. The photos were captured with an inverted optical microscope (Leica DMI3000B/Nikon Digital Camera Dxm1200F).

### 2.4 Isolation and cultivation of porcine umbilical vein derived-endothelial cells (pUVEC)

Porcine umbilical cords were procured from swine fetuses at 72–80 days post-insemination. The cells were isolated from the umbilical vein by enzymatic digestion with 0.05% trypsin/0.02% EDTA, following the protocol of Davis and co-workers (Davis et al., 2007). After locating the umbilical vein, a 20-gauge Surflo IV catheter (Terumo, Japan) was inserted and secured with 2–0 silk suture. The lumen was perfused with 20 mL pre-warmed Hank’s balanced salt solution (HBSS) to remove residual blood. The distal end of the vein was then clamped with a hemostat and a second HBSS flush confirmed vessel integrity. The syringe was replaced with 10 mL of 0.05% trypsin/0.02% EDTA, which was infused until it exited the distal opening; this end was immediately ligated with 2–0 silk, leaving the vein completely filled with the enzymatic solution. The cord—with catheter and syringe still attached—was incubated in PBS at 37°C for 15 min. After incubation, the distal ligature was cut over a 50 mL conical tube, the digestion mixture was expelled, and the vein was flushed once more with HBSS to recover remaining cells. Detached porcine umbilical-vein endothelial cells (pUVECs) were pelleted (1,200 × g, 5 min) and resuspended in porcine endothelial growth medium (pEGM; Cell Applications, United States) for subsequent culture. After isolation, the cells were seeded at 15,000 cells/cm^2^ in 152 cm^2^ Petri dishes coated with 1% (v/v) gelatin in porcine endothelial growth medium (pEGM) (Cell Applications, United States) and incubated at 37°C in a humidified atmosphere of 5% CO_2_. Culture medium was replaced after 48 h from the initial seeding and changed every 2 days. Once confluent (100%), cells were either frozen or sub-cultured following cell detachment with 0.05% trypsin/0.02% EDTA and seeded between 3,000 and 6,000 cells/cm^2^ in 152 cm^2^ Petri dishes coated with 1% (v/v) gelatin with pEGM and incubated at 37°C in a humidified atmosphere of 5% CO_2_. The medium was changed every 3–4 days. Generally, passage 5–8 cells were used in the experiments. The pictures were captured with an inverted optical microscope (Leica DMI3000B/Nikon Digital Camera Dxm1200F).

### 2.5 Phenotypical characterization of pMSC(M), pASMC and pUVEC

Flow cytometry (FC) and immunofluorescence assays were employed to evaluate the expression of a panel of specific markers for pMSC(M), pASMC, and pUVEC. ChromPure Swine IgG, whole molecule (Jackson ImmunoResearch Europe, Ltd., United Kingdom) served as the Fc blocking reagent for FC studies, adhering to the manufacturer’s instructions. The antibodies validated by us for cell characterization are listed in Table 1. A minimum of 10,000 events were collected for FC, and cells were analyzed on a BD FACS Calibur flow cytometer using CellQuest Software (Becton Dickinson, United States). All data were analyzed using FlowJo Software (FlowJo, Becton Dickinson, United States).

**TABLE 1:** List of the distinct antibodies used for the immunophenotypically characterization of pMSC(M), pASMC, and pUVEC.

Cell type	Analytical method	Antibodies
pMSC(M)	Flow Cytometry	Primary Antibodies
		FITC-conjugated anti-CD11b (Clone: ICRF44, Biolegend, United States)
		FITC-conjugated anti-CD45 (Clone: K252.1E4, Bio-Rad, United States)
		PE-conjugated anti-CD14 (Clone: M5E2, Biolegend, United States)
		PE-conjugated anti-CD29 (Clone: TS2/16, Biolegend, United States)
		PE-conjugated anti-CD31 (Clone: LCI-4 Bio-Rad, United States)
		Alexa Fluor 488-conjugated anti-CD44 (Clone: IM7, Biolegend, United States)
		PerCP-Cy5.5 conjugated anti-CD90 (Clone: 5E10, Biolegend, United States)
		Anti-CD34 (Polyclonal, bs-8996R, Bioss Antibodies, United States)
	Immunofluorescence	Primary Antibodies
		PE-conjugated anti-CD29 (Clone: TS2/16, Biolegend, United States)
		Anti-CD44 (Clone: IM7, StemCell Technologies, United Kingdom)
		Anti-CD90 (Clone: 5E10, StemCell Technologies, United Kingdom)
pASMC	Flow Cytometry	Primary Antibodies
		Same as pMSC(M), except
		Anti-CD144 (Clone: F-8, Santa Cruz Biotechnology, United States)
		Anti-SM22α (Clone: TAGLN/247, Novus-Biologicals, Bio-Techne, United Kingdom)
	Immunofluorescence	Primary Antibodies
		Same as pMSC(M), except
		Anti-SM22α (Clone: TAGLN/247, Novus-Biologicals, Bio-Techne, United Kingdom)
		Anti- αSMA (Clone: SP171, Sigma-Aldrich, Germany)
		Caldesmon (Clone: C21, Santa Cruz Biotechnology, United States)
pUVEC	Flow Cytometry	Primary Antibodies
		PE-conjugated anti-CD29 (Clone: Ts/16, Biolegend, United States)
		PE-conjugated ant-CD31 (Clone: LCI-4, Bio-Rad, United States)
		Alexa Fluor 488-conjugated anti-CD44 (Clone: IM7, Biolegend, United States)
		Anti-CD34 (Polyclonal, bs-8996R, Bioss Antibodies, United States)
		Anti-CD105 (Clone: MEM-229, Novus-Biologicals, Bio-Techne, United Kingdom)
		Anti-CD144 (Clone: F-8, Santa Cruz Biotechnology, United States)
	Immunofluorescence	Primary Antibodies
		Anti-CD31 (Clone: H-3, Bioss Antibodies, United States)
		Anti-CD44 (Clone: IM7, StemCell Technologies, United Kingdom)
		Anti-CD105 (Clone: MEM-229, Novus-Biologicals, Bio-Techne, United Kingdom)
		Anti-CD144 (Clone: F-8, Santa Cruz Biotechnology, United States)
		Anti-Tie-2 (Clone: C-20, Santa Cruz Biotechnology, United States)
		Anti-Vimentin (Clone: V9, Santa Cruz Biotechnology, United States)

The FC data of pMSC(M), pASMC, and pUVEC were confirmed by immunofluorescence staining in 24-well plates (ThermoFisher Scientific, United States). pMSC(BM) were seeded at 3,000 cells/cm^2^ in 24-well plates coated with 0.2% (v/v) gelatin, and the cell culture medium was changed every 5 days until reaching confluence (80%–90%). pASMC were seeded at 6,000 cells/cm^2^ in 24-wells plates coated with 0.2% (v/v) gelatin, and the cell culture medium was changed every 5 days until reaching confluence (80%–90%). Finally, pUVEC were seeded at 4,000 cells/cm^2^ in 24-wells plates coated with 1% (v/v) gelatin, and the cell culture medium was changed every 5 days until reaching confluence (100%). All the cells were cultured at 37°C in a humidified atmosphere of 5% CO_2_. Then, the cells were fixed for 20 min at room temperature with 4% (v/v) paraformaldehyde (PFA) (ThermoFisher Scientific, United States), and stained with primary antibodies (Table 1) and appropriate secondary antibodies. The photos were captured with a fluorescence microscope (Leica DMI3000B/Nikon Digital Camera Dxm1200F) and processed using ImageJ 1.51s software.

### 2.6 Functional characterization of pMSC(M)

To further characterize pMSC(M), we evaluated their differentiation potential using commercial differentiation kits following manufacturer’s instructions (StemPro Adipogenesis Differentiation Kit, StemPro Osteogenesis Differentiation Kit, StemPro Chondrogenesis Differentiation Kit, all from ThermoFisher Scientific, United States). Briefly, for adipogenic, osteogenic and chondrogenic differentiation assays the cells were cultured with specific differentiation media and kept in culture for 21 days. The differentiation was assessed by Oil Red O, alkaline phosphatase (ALP)/Von Kossa and alcian blue staining’s (for adipogenic-, osteogenic-, and chondrogenic differentiation, respectively). The photos were captured with an inverted optical microscope (Leica DMI3000B/Nikon Digital Camera Dxm1200F).

### 2.7 Functional characterization of pUVEC

Finally, to evaluate the functional activity of pUVEC, the cells were cultured in 8-wells chamber slides (Nunc Lab-Tek II Chamber Slide, ThermoFisher Scientific, United States) at 30,000 cells/well and incubated at 37°C in a humidified atmosphere of 5% CO_2_ for 2 days. Then, the cells were treated with a Dil-Ac-LDL staining kit (Cell Applications, United States), following the manufacturer’s instructions. The photos were captured with a fluorescence microscope (Leica DMI3000B/Nikon Digital Camera Dxm1200F) and processed using ImageJ 1.51s software.

### 2.8 Liver harvesting, decellularization and acellular liver discs preparation

The procedure for liver harvesting, decellularization, and disc preparation was adapted from the protocol described by Baptista et al. in ferret and porcine livers [3], [22]. Here, livers were harvested from cadaveric rats after a freeze–thaw cycle, which preserved the native vascular architecture. Following that, portal vein (PV) and hepatic artery (HA) were cannulated and perfused with 2L of distilled water, 4L of decellularization solution composed of 1% Triton X-100 (Sigma-Aldrich, Germany) and 0.1% ammonium hydroxide (Sigma-Aldrich, Germany) and, finally, with 8L of distilled water to wash out the decellularization solution from the tissue. To obtain liver discs, decellularized livers were cut into wedges, embedded in optimal cutting temperature compound (OCT compound) (Sigma-Aldrich, Germany) in tissue cryomolds (Tissue-Tek Cryomolds, ProSciTech, Australia) and flash-frozen with liquid nitrogen. These cryopreserved liver lobes were mounted onto a cryotome (Leica CM 1950, Leica, Germany) to obtain liver ECM sections of 100 µm thickness. An 8 mm diameter biopsy punch (Thermofisher Scientific, United States) was used to generate discs from decellularized liver sections before placing them in a 48-well cell culture-treated plate as we illustrated in the representative Figure 4A. Then, after multiple washes with PBS, the discs were sterilized by UV irradiation and stored at 4°C in sterile PBS. Additionally, to assess the suitability of the decellularized matrices for cell culture post-decellularization, the residual DNA content in the disc dECM was quantified using an extraction kit (Qiagen, Germany) and H&E staining’s (Figure 4B). Finally, key structural proteins presence was evaluated by Alcian blue, Masson and immunofluorescence tissue staining’s (Figures 4B,C) using the antibodies listed in Table 2.

**TABLE 2:** List of antibodies used for the immunofluorescence analysis of decellularized ECM discs (dECM-discs).

Antibody	Clone	Manufacturer	Concentration
Col I	Polyclonal	SouthernBiotech, United States	1:100
Col III	Polyclonal	SouthernBiotech, United States	1:100
Col IV	Polyclonal	SouthernBiotech, United States	1:100
Laminin	Polyclonal	Abcam, United Kingdom	1:100

### 2.9 Transfection of pUVEC with GFP lentiviral vectors

pUVEC were cultured in expansion conditions and once 80%–90% confluent, the cells were infected with Lenti-Green Fluorescent Protein (Lenti-GFP) vectors (prepared in our lab), expanded for a week, and finally sorted through fluorescence-activated cell sorting (FACS Aria, Beckton Dickinson, United States) to separate labelled cells from unlabeled cells.

### 2.10 pUVEC-GFP, pASMC and pMSC(M) seeding on acellular ECM discs (3D culture)

Sterilized dECM discs were incubated with MCDB 131 medium (Gibco, Thermofisher Scientific, United States) supplemented with 5% FBS + 2 mM L-Glutamine, 1% (v/v) P/S, 5 μg/mL Insulin (Sigma-Aldrich, Germany), 10 μg/mL Transferrin (Sigma-Aldrich, Germany), 50 ng/mL vascular endothelial growth factor (VEGF), 40 ng/mL epithelial growth factor (EGF), 40 ng/mL fibroblast growth factor (FGF-2) and 40 ng/mL insulin-like growth factor (IGF-1) (all growth factors from Peprotech, United Kingdom) at 37°C, 5% CO_2_ the day before cell seeding and then removed before cell seeding. Considering the physiological cellular composition of the vascular network, we seeded 1 × 10^6^ cells (80% pUVEC, 15% pMSC(M) and 5% pASMC) onto acellular dECM discs and 48-wells cell culture-treated plates (2D control). Cells were resuspended in 30 µL of EC medium for the seeding onto each disc. The cell suspension was slowly pipetted on top of each disc and incubated for 30 min at 37°C, 5% CO_2_ to enhance cell attachment before adding additional volume of culture medium. As a 2D control, the same number of pUVEC, pASMC and pMSC(M) were seeded in a 0.2% (v/v) gelatin-coated 48-well tissue culture-treated plate. The culture medium was changed every 12 h during the first 48 h to remove excess non-adherent cells and then every 2 days. Discs were kept in culture for up to 7 days for immunofluorescence analysis, being monitored by inverted microscopy at days 3–7, assessing GFP expression.

### 2.11 Culture media assays

To determine pUVEC functional activity, the conditioned medium from 3D and 2D cultures was collected daily and stored at – 80°C for further analysis (n = 3). Then, the total nitric oxide (NO) production was measured in triplicate using BioVision’s Nitric Oxide Colorimetric Assay Kit (BioVision, United States) using a microplate reader (SynergyHT, Biotek Instruments, United States) following the manufacturer’s instructions.

### 2.12 Immunohistochemical analysis

After 7 days, recellularized discs and 2D controls were prepared for immunofluorescence analysis (Table 3). Thus, dECM discs were fixed for 24 h in 4% PFA, tissue processed, and paraffin-embedded for histological analysis. 5 μm sections were cut from the embedded paraffin blocks using a Leica microtome (Leica Biosystems, Germany) and placed onto microscopy slides. For immunofluorescence analysis, the slides were deparaffinized, and the antibody target retrieval was carried out using Target Retrieval Solution (Dako, United States) following the manufacturer’s instructions. Then, the slides were treated with 1% sodium borohydride in PBS and blocked for 1 h using serum-free protein block (Agilent Dako, United States) with 0.1% Saponin for intracellular staining permeabilization. The slides were incubated overnight at 4°C with primary antibodies diluted in the above-mentioned serum-free protein block (Table 3). The next day, the slides were washed with 1X Tris-buffered saline with 0.05% Tween-20 (TBST) (Sigma-Aldrich, Germany) to remove unbound primary antibodies and reduce nonspecific background. Tween-20 helps prevent nonspecific protein interactions, while the Tris buffer maintains physiological pH and ionic strength in the tissue. After that, discs were incubated with the appropriate secondary antibodies (Invitrogen, Thermofisher Scientific, United States) diluted in serum-free protein block for 1 h at room temperature (RT). Then, the slides were washed with 1X TBST again and cover-slipped with Fluoromount-G mounting medium (Thermofisher Scientific, United States) containing 1.5 μg/mL DAPI (Sigma-Aldrich, Germany). The images were captured with an Olympus IX81 Widefield Fluorescent Microscope and a confocal LSM 880 microscope (Carl Zeiss, Germany).

**TABLE 3:** List of antibodies used for the immunofluorescence analysis of cell-seeded ECM discs.

Antibody	Clone	Manufacturer	Concentration
CD31	SZ31	Dianova, Biozol, Germany	1:100
SM-MHC	SPM201	MyBiosource Inc., United States	1:100
CD73	Polyclonal	Novus-Biologicals, Bio-Techne, United Kingdom	1:100

### 2.13 Statistics

Data are presented as mean ± standard deviation (SD). Normality of the datasets was assessed before analysis. Comparisons between control 2D and liver disc groups at each time point were performed using unpaired two-tailed T-tests. Differences were considered statistically significant when p < 0.05. Statistical analyses were conducted using Graphpad Prism v9 (Graphpad Software, Inc., United States).

## 3 Results

### 3.1 pMSC(M): isolation, culture, phenotypical and functional characterization

**FIGURE 1:**
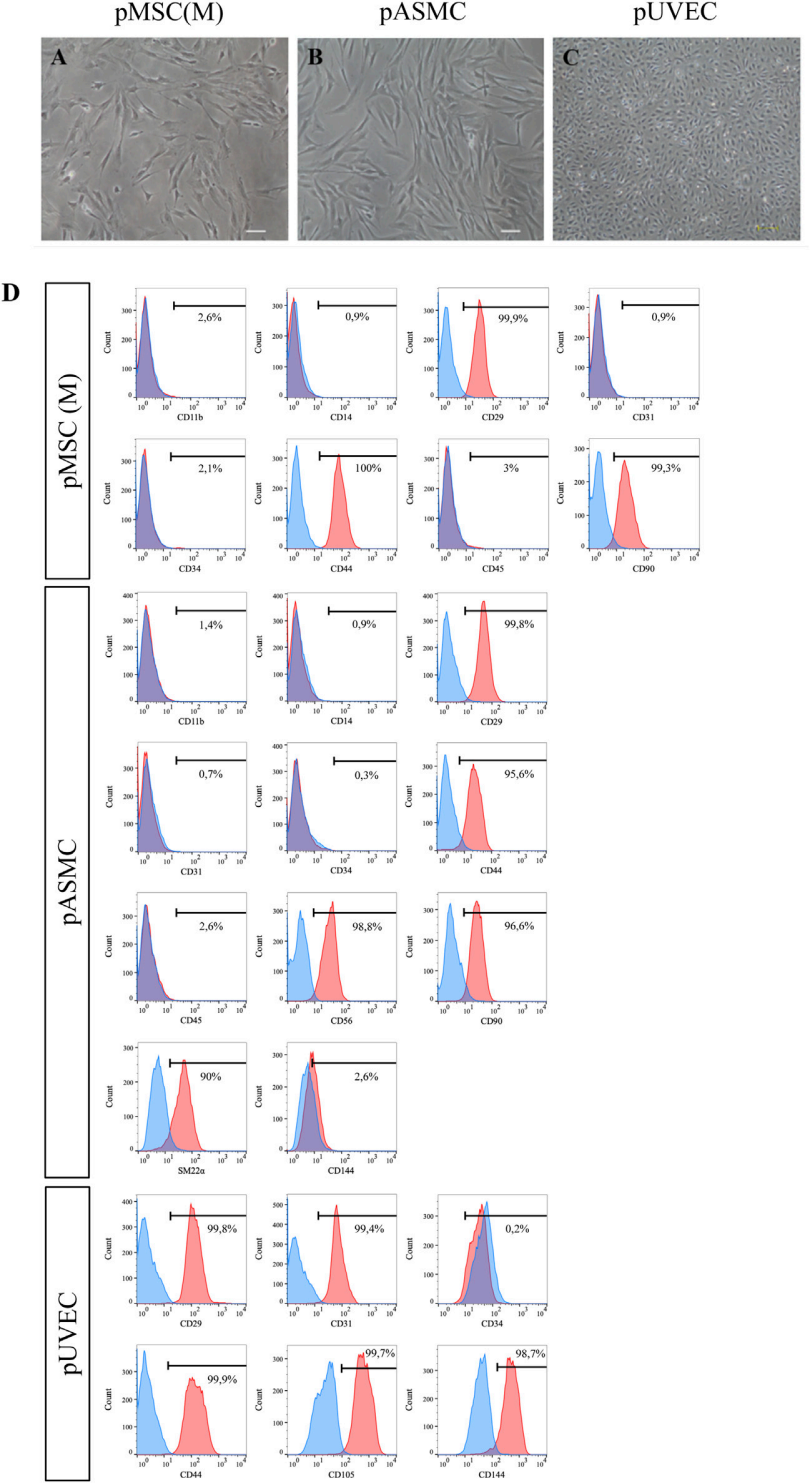
Morphology of pMSC(M), pASMC, and pUVEC. **(A)** Microscopic image of early passage pMSC(M) (passage 1). Scale bar: 100 µm. **(B)** Morphology of pASMC 8 days after the initial seeding, after isolation (passage 0). Scale bar: 100 µm. **(C)** Morphology of pUVEC isolated from porcine umbilical vein (passage 1). Scale bar: 200 µm. **(D)** Phenotypical characterization of pMSC(M), pASMC and pUVEC by flow cytometry. A strong positive expression of MSC, SMC, and EC markers was shown, indicating that the cells isolated using our methods possess typical MSC, SMC, and EC immunophenotypically features.

The MNC fraction, extracted from porcine BM, was cultured in DMEM/FBS-MSC culture medium following isolation. Distinct pMSC(M) colonies became visible 3–5 days after the initial seeding, with an elongated fibroblast-like morphology, which was more prominent in cells organized within colonies, as previously reported (Ringe et al., 2002; Noort et al., 2012). The cultures were heterogeneous, consisting of different subpopulations of cells, including small polygonal cells interspersed with typical spindle-shaped cells (Figure 1A), like observations made with human MSC(M) (Sekiya et al., 2002; Smith et al., 2004; Haasters et al., 2009). The number and size of the colonies progressively increased, reaching confluence (80%) within 10–12 days after the initial seeding.

**FIGURE 2:**
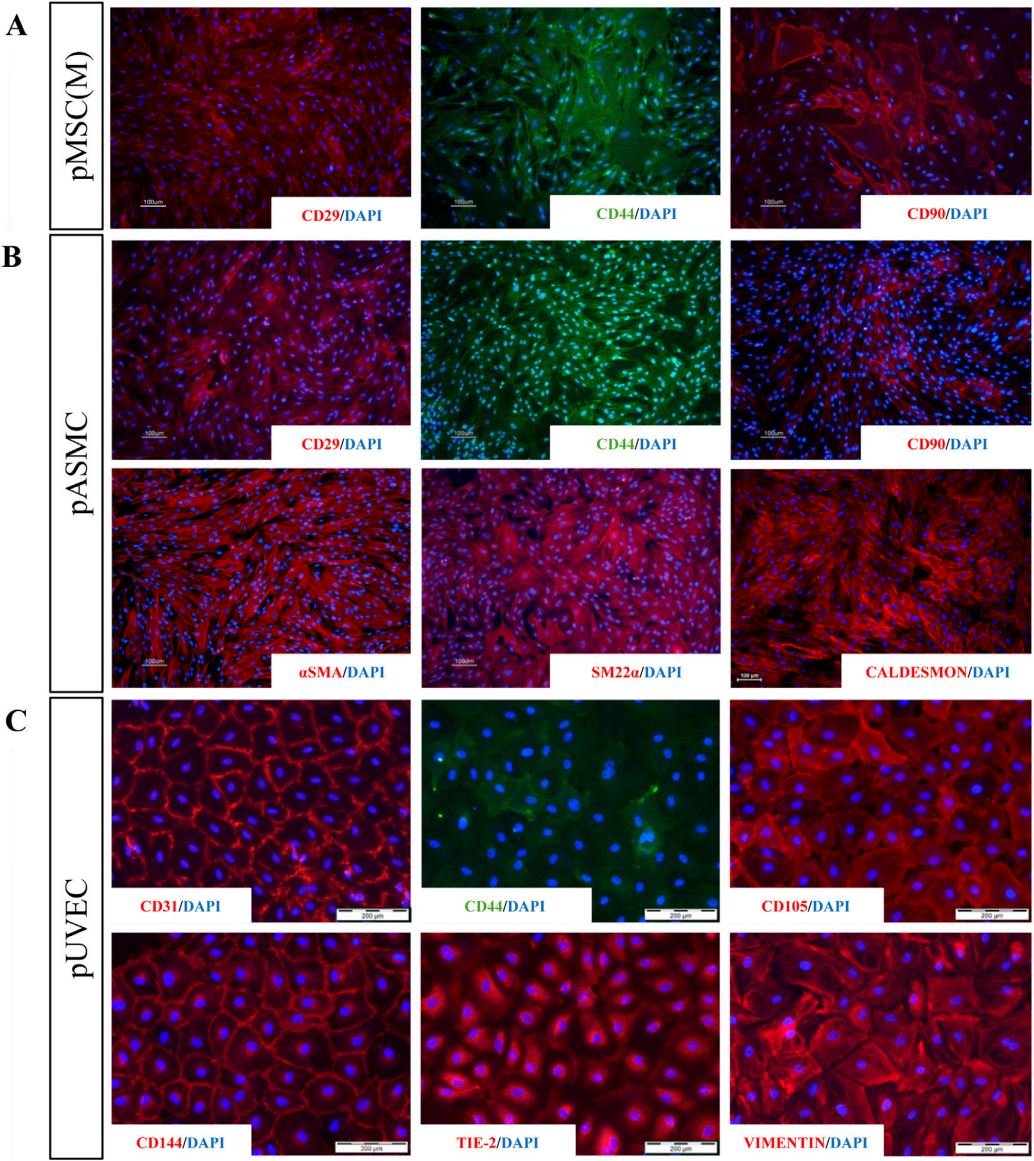
Immunofluorescence analysis for a panel of MSC, SMC, and EC markers. **(A)** Staining for extracellular markers of pMSC(M) (CD29, CD44, CD90) showing positive expression for the three characteristic MSC markers. **(B)** Staining for extracellular (CD29, CD44, CD90) and intracellular (aSMA, SM22a, Caldesmon) markers of pSMC indicating that SMC derived from porcine aorta possess relevant phenotypical features of SMC (aSMA, SM22a, Caldesmon) and a contractile phenotype (showed by the strong expression of SM22a and Caldesmon). **(C)** Staining for specific EC markers (CD31, CD44, CD105, CD144, Tie-2, Vimentin). The expression of all EC-specific markers demonstrated that ECs isolated from the porcine umbilical vein express representative features of vascular ECs, including four of the most widely used EC-specific markers (CD31, CD105, CD144, Tie-2). Scale bar: 100 µm and 200 µm.

The immunophenotypic features of pMSC(M) were assessed by FC using a panel of surface markers. This panel include CD90, a marker representative of the mesenchymal cell lineage (Dominici et al., 2006); additional markers typically expressed by MSC (CD29, CD44); and exclusion lineage markers (CD11b, CD14, CD31, CD34, CD45) which are specific to non-mesenchymal cell types such as endothelial and hematopoietic cells. pMSC(M) consistently expressed CD29, CD44, and CD90, while the expression of endothelial (CD31) and hematopoietic markers was absent (Figure 1D). The expression of CD29, CD44, and CD90 was further confirmed by immunofluorescence (Figure 2A), supporting the mesenchymal phenotype of these cells by qualitative observation.

**FIGURE 3:**
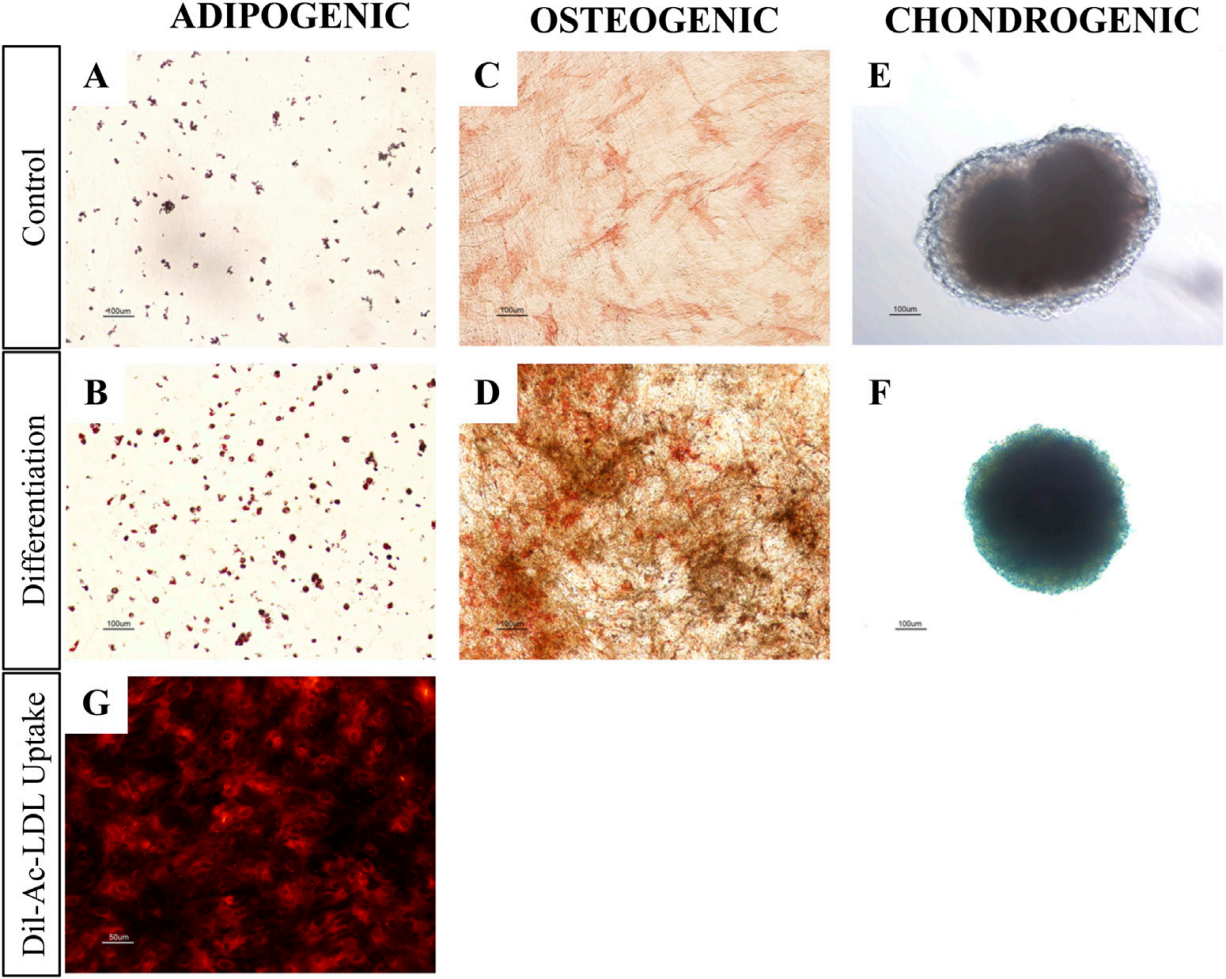
pMSC(M) and pUVEC functional assays. **(A-F)** Adipogenic, osteogenic and chondrogenic differentiation of pMSC(M). Controls were cultured using an expansion medium. Differentiation was induced using commercially available kits from Thermofisher Scientific. **(A, B)** Oil red O staining for adipogenic differentiation. The slight staining of the control is probably due to the spontaneous differentiation of porcine MSC when cultured for prolonged time at confluency. The intensity of Oil Red O staining of pMSC(M) cultured with differentiation medium was significantly higher, allowing for the detection of the characteristic intracellular lipid vesicles of mature adipocytes (red staining). **(C, D)** ALP/Von Kossa staining for osteogenic differentiation. pMSC(M) cultured with expansion medium only showed a minor red staining induced by ALP. pMSC(M) cultured with differentiation medium differentiated as indicated by the presence of black stains (calcium deposits) and a distinguishable and intense red staining, which suggests the presence of osteoblasts in the culture. **(E, F)** Alcian blue staining for chondrogenic differentiation. pMSC(M) of the control culture not differentiated, as shown by the total absence of blue staining. Cells cultured with differentiation medium differentiated to chondrocytes as demonstrated by the dark blue staining, which suggests the presence of glycosaminoglycans of the cartilage. Scale bars: 100 µm. **(G)** Dil-Ac-LDL uptake assay of pUVEC cultured with pEGM culture medium. The intense red staining indicates that pUVEC were able to internalize Dil-Ac-LDL, a typical function of endothelial cells. Scale bar: 50 µm

To evaluate their differentiation potential, we tested the trilineage capacity of pMSC(M) torwards adipogenic, osteogenic, and chondrogenic lineages. Adipogenic differentiation was evaluated by Oil Red O staining (Figures 3A,B), confirming the formation of lipid droplets, although the extent of differentiation was modest. This limited adipogenic response may be attributed to the use of the StemPro™ Adipogenesis Differentiation kit, which is optimized for human MSC and may lack specific factors required to promote efficient adipogenesis in pMSC. Osteogenic differentiation was evaluated by ALP/Von Kossa staining (Figures 3C,D), showing that pMSC cultured in osteogenic differentiation medium exhibited strong ALP activity and calcium deposition, as evidenced by the presence of intense red and black staining, respectively, indicative of osteogenic commitment. Chondrogenic differentiation was analysed using Alcian Blue staining (Figures 3E,F), which reveled dark blue-stained cells consistent with glycosaminoglycan deposition, a fundamental component of cartilage. In summary, pMSC(M) presented relevant mesenchymal features, including the expression of canonical mesenchymal surface markers (CD29, CD44, and CD90) and the ability for trilineage differentiation into adipocytes, osteoblasts, and chondrocytes *in vitro*.

### 3.2 pASMC: isolation, culture, and phenotypical characterization

The cultured primary pASMCs were attached efficiently to the culture dishes and the selected pASMC population had a spindle-shaped fibroblast-like appearance (Figure 1B). The number and size of the colonies increased progressively to reach confluence (90%) 10–12 days after the initial seeding.

To define the immunophenotypically features of pASMC, we evaluated by FC the expression of markers that have been described as representative of smooth muscle cells (SM22*α*) (Rensen et al., 2007), other antigens generally expressed by MSC (CD29, CD44, CD90), and lineage markers (CD11b, CD14, CD31, CD34, CD45, CD56, CD144). Essentially, all pASMC expressed SM22*α*, CD29, CD44, CD56, and CD90. However, the expression of the endothelial markers CD31 and CD144, as well as the lineage markers, was absent (Figure 1D). Moreover, we confirmed the expression of some of these markers by immunofluorescence, including more typical smooth muscle markers (SM22*α*, *α*SMA, and Caldesmon) and other antigens commonly expressed by SMC (CD29, CD44, CD90) (Figure 2B). Notably, the most expressed markers were the contractile SMC markers SM22*α* and *α*SMA, reported as contractile phenotype-associated markers in SMC (Rensen et al., 2007). Thus, considering both sets of data, we showed that the isolated SMC from the porcine aorta showed phenotypical features characteristic of contractile SMC.

### 3.3 pUVEC: isolation, culture, phenotypical and functional characterization

The primary pUVEC were selected by their adhesive properties, and cell cultures were heterogeneous and constituted by different subpopulations of cells, including cobblestone-like shaped cells and elongated cells (Figure 1C). To define the immunophenotypic profile of pUVEC, we evaluated the expression of representative markers for endothelial (CD31, CD105, CD144) (Post et al., 2013), mesenchymal (CD29, CD44), and progenitor or microvascular cells (CD34) by FC. Essentially, all pUVEC expressed CD29, CD31, CD44, CD105, and CD144, with lack of expression of CD34 (Figure 1D). Moreover, we confirmed by immunofluorescence the expression of specific endothelial markers (CD31, CD105, CD144, Tie-2), other antigens commonly expressed by EC (CD44), and vimentin, an intermediate filament of the cytoskeleton which plays a critical role in the physiological endothelial mechano-response and inherent to the endothelial phenotype (Helmke et al., 2001; Conway et al., 2013). Notably, the most common markers for EC (CD31, CD105, CD144, and Tie-2) and vimentin were highly expressed by pUVEC (Figure 2C). To further demonstrate the endothelial phenotype of pUVEC and their function, we tested their capacity to internalize DiI-Ac-LDL (Voyta et al., 1984). The DiI-Ac-LDL uptake analysis showed that pUVEC significantly internalized the dye, as demonstrated by the associated red fluorescence detectable using a conventional fluorescence microscope (Figure 3G). These combined results demonstrated that pUVEC isolated from porcine umbilical vein showed a typical endothelial phenotype.

### 3.4 Co-cultured cell functionality in a liver dECM microenvironment disc

**FIGURE 4:**
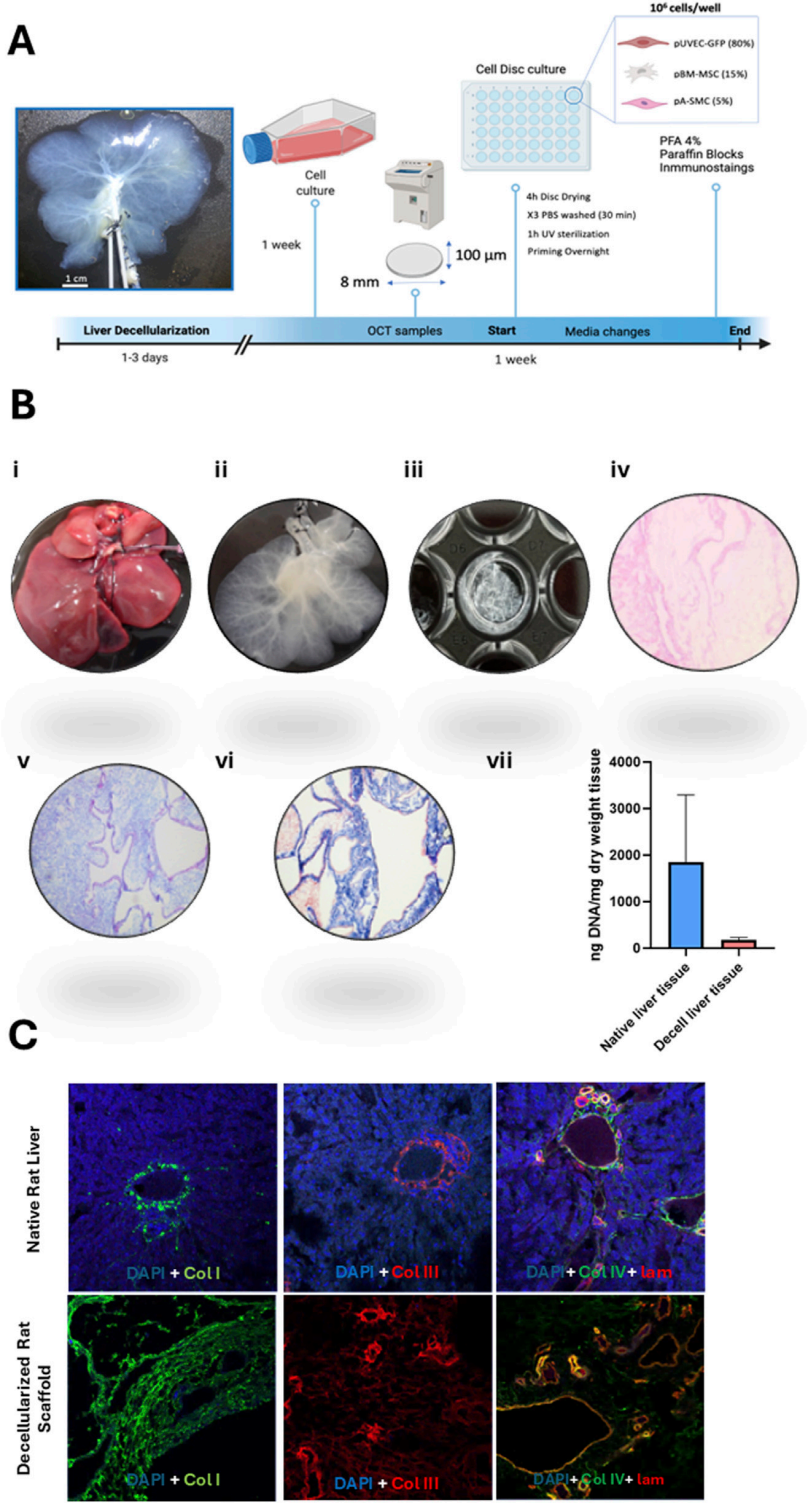
Overview of decellularized matrix disc generation and characterization. **(A)** Step-by-step guide to the generation of 3D liver decellularized matrix discs in 48-wells cell culture-treated plates. Following liver decellularization, the matrix was processed to obtain dECM discs. Concomitantly, the cells were cultured under conventional static conditions. These discs were subsequently seeded with the porcine vascular cells, maintained in culture for a week, and then analyzed by immunohistochemistry. **(B)** Histological characterization of the decellularized matrix discs: (i) The process begins with the isolation of the native rat liver, followed by (ii) its decellularization to remove cellular components. (iii) Afterward, 100 µm-thick decellularized hepatic matrix discs are created. (iv) Hematoxylin and eosin (H&E) staining of the decellularized discs confirms the absence of cellular nuclei, while (v) Alcian blue staining highlights the presence of glycosaminoglycans (GAGs) in the extracellular matrix. (vi) Masson’s trichrome staining reveals muscular fibers in red and collagen fibers in blue. Images taken at 10x magnification. (vii) Quantification of residual DNA content expressed as ng/mg dry weight tissue. DNA levels in decellularized liver (ng/mg) were compared to those in native rat liver tissue (ng/mg). **(C)** Immunofluorescence analysis of the main extracellular matrix proteins: Collagen I (green), Collagen II (red), and a combination of Collagen IV (green) and Laminin (red), which appear yellow/orange upon merging. Nuclei are stained in blue (DAPI). Images captured at 10x magnification.

Decellularized extracellular matrix (dECM) presents a minimal risk of immune rejection, as its immunogenic protein components are largely removed. Instead, the primary source of rejection arises from residual cellular content. In this study, we utilized decellularized rat liver matrices, which not only maintain the vascular tree architecture for porcine cell repopulation but also offer an advantage due to their smaller size, facilitating efficient decellularization and subsequent recellularization. Furthermore, since ECM protein composition is highly conserved across species, these matrices serve as a reliable model (Caralt et al., 2014; Ferreira et al., 2020). Thus, a more detailed characterization of the extracellular matrix was performed following the decellularization process to verify its suitability for cell culture (Figure 4B). To this end, first the quantification of residual DNA was assessed and the presence of structural components in the extracellular matrix, such as collagen (blue) and muscular fibers (red) (Figure 4Bvi) along with glycosaminoglycans (GAGs) (Figure 4Bv), were confirmed through Masson’s trichrome and Alcian blue staining, respectively. After the decellularization protocol, DNA concentration in the scaffold was reduced by 93.3% (176, 60 ng/mg ± 53, 30 SD) compared to native liver (2,594 ng/mg ± 914, 56 SD) (Figure 4Bvii). This result was confirmed by the absence of cellular nuclei, as evidenced by H&E staining of the processed sections (Figure 4Biii). Meanwhile, key structural proteins such as Collagen I, Collagen III, Collagen IV, and Laminin, which delineate hepatic vascular regions and provide structural support to the matrix, were preserved following detergent-based cell removal. The absence of DAPI staining in the decellularized tissue indicates the success of the decellularization process (Figure 4C).

**FIGURE 5:**
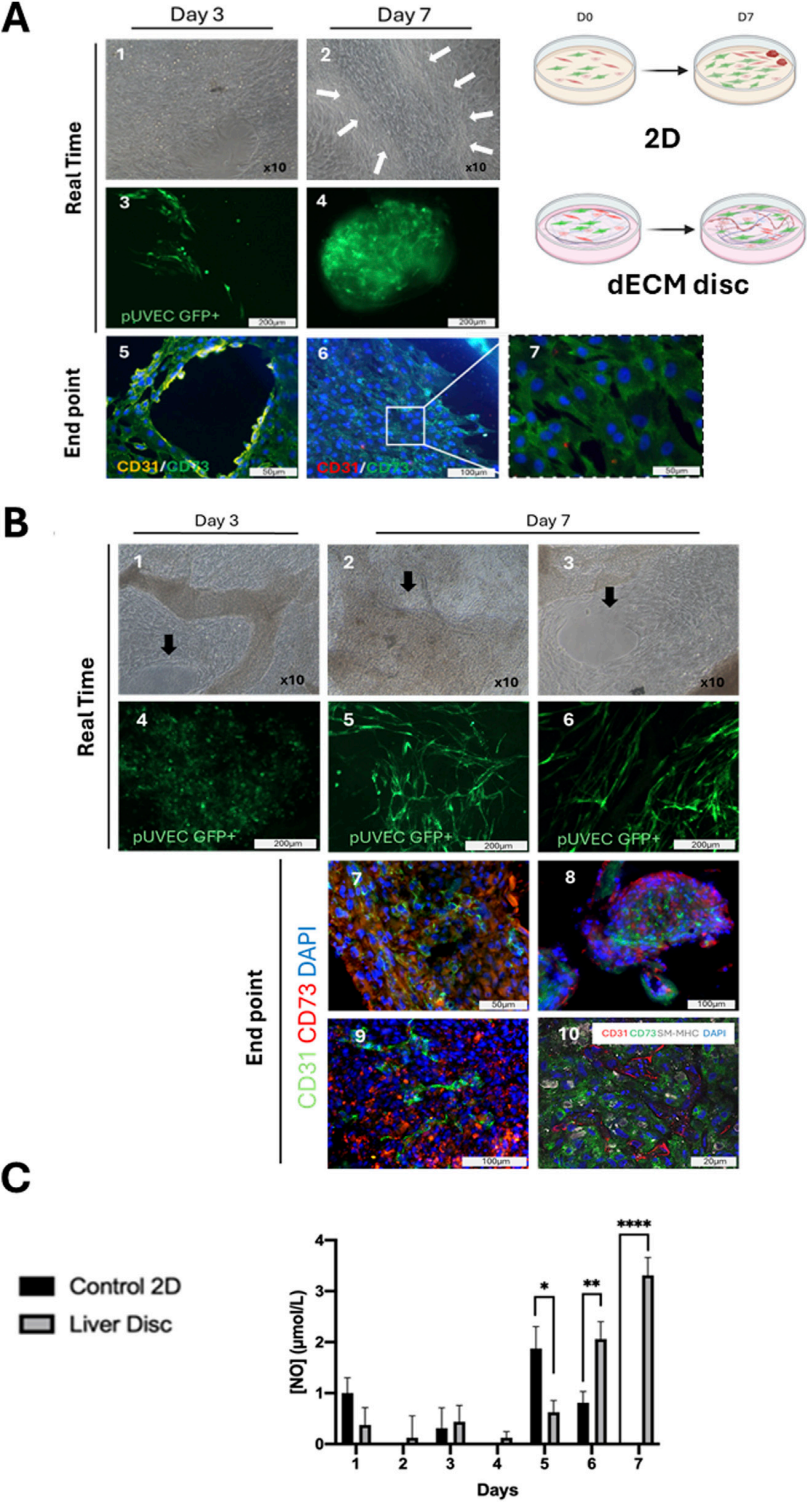
Vascular cells in co-culture in a 3D liver dECM microenvironment. **(A)** 2-D culture control monitoring: A.1, A.2) Phase contrast images of cell seeded-2D control at day 3 and 7 of culture. The different cell types grew, forming compact multilayers on day 3 of culture, whereas by day 7, stromal cells formed a monolayer, clustering other cell groups into small islets (arrows) and triggering a loss of confluence due to contact inhibition. Microscopy magnification: x10. A.3, A.4) Native expression of GFP+ pUVEC confirms the displacement of the endothelial cells from day 3, where they were dispersed in the monolayer, to day 7, where they are confined to the periphery of the plate. A.5, A.6, A.7) Immunofluorescence analysis of cell seeded in 2D control demonstrates how the aligned endothelial cells (CD31+, yellow), initially interacting with mesenchymal cells (CD73+, green) on day 3, lose their architectural organization and become displaced by the mesenchymal cells over time. **(B)** Seeded-dECM liver discs monitoring: B.1, B.2, B.3) Phase contrast images of cell seeded-liver dECM disc at day 3 and 7 of culture. Cells cultured onto dECM discs colonized the decellularized matrix and proliferated in multilayers, maintaining the confluence until the end of the culture period. Arrows highlight structurally organized regions where endothelial cells aligned to form vascular-like structures. B.4, B.5, B.6) Through the expression of GFP+ pUVEC in real-time we detected a higher cell proliferation since day 3 of culture when cells were cultured onto dECM discs compared to the 2D control. On day 7, the staining is even more intense for cells cultured on dECM discs, showing a distinct cell spatial organization in microvessels. Scale bar: 100 and 200 µm. B.7, B.8, B.9) Immunofluorescence analysis of cell-seeded-dECM liver discs at day 7 of culture, showing microvascular structures constituted by CD31+ pUVEC with CD73+ pMSC(M) surrounding them. B.10) Confocal microscopy image of endothelial, mesenchymal and smooth muscle cell markers (CD31, CD73 and SM-MHC, respectively) at day 7 of culture, revealing that dECM has a notable influence on cell spatial distribution, enhancing the formation of microvascular structures in the acellular liver discs. Scale bars: 20 and 100 µm. **(C)** Daily measurement of NO concentration released in the cell culture medium for 2D control and cell-seeded liver dECM discs. Results are presented as mean and ± SD (n=3). A significant difference in NO release was observed for EC cultured onto dECM discs, especially at the end of the culture period, indicating that the cells maintained their viability and functionality, whereas the EC cultured under static conditions were less or not functional at the end of the culture.

After conducting multiple co-culture experiments with porcine vascular cells, significant differences were observed in cellular organization between the 2D controls (Figure 5A) and the cell seeded-dECM discs (Figure 5B) in 3 and 7 days of culture. Phase contrast microscopy showed over-confluent cells in culture dishes with hardly discernable phenotypes at 3 and/or 7 days of culture (Figures 5A1,A2). Likewise, robust cellular proliferation was observed on the dECM discs and surrounding plastic (Figures 5B1–B3). However, fluorescence microscopy imaging of cell-seeded dECM liver discs revealed a progressive intercellular association and many structures resembling microvascular formations constituted by the GFP^+^ pUVEC at day 7 of culture (Figures 5B5,B6), which were not detected at day 3 of culture (Figure 5B4). In contrast, in 2D controls, most GFP + pUVEC were gradually displaced by other cells in culture over time to the sides of the culture’s dishes. By day 3, GFP + pUVEC were intermixed with other cells, appearing only in a few discrete, isolated EC clusters (Figure 5A3). Though by day 7, these cells were further displaced by the expansion of larger cytoplasmic cells, failing to integrate into the monolayer and instead forming 3D cellular aggregates at the periphery of the plate wells, with no obvious microvascular organization (Figure 5A4).

Then, immunofluorescence analysis of the cell-seeded discs showed the formation of linear and branched microvascular tubular structures constituted by CD31^+^ pUVEC with CD73^+^ pMSC(M) surrounding these throughout the whole dECM disc (Figures 5B7–B9). In contrast, in 2D controls, we could only observe how the spatial cellular organization observed on day 3, where endothelial cells were able to moderately arrange alongside mesenchymal cells (Figure 5A5), disappeared by day 7, leaving only a CD73^+^ cell monolayer (pMSCs) at the culture dish periphery with some interspersed CD31^+^ cells (pUVECs) (Figures 5A6,A7). Finally, cell-seeded discs results were further confirmed with confocal microscopy imaging that showed the presence of microvascular-like structures composed of CD31^+^ cells (pUVEC) with surrounding CD73^+^ cells (pMSC(M)) and SM-MHC^+^ cells (pASMC) (Figure 5B10). Considering these results, it can be observed that the cellular spatial localization in cell-seeded dECM discs may resemble the physiological arrangement within capillaries (internal diameter < 10 µm), but not in larger vascular structures. Specifically, this consists of a single layer of EC forming the microvasculature, surrounded by CD73^+^ pericytes (basal lamina) and, to a lesser extent, by smooth muscle cells, which don’t seem to organize in a tunica media.

Daily measurements of nitric oxide (NO) concentrations in the supernatant were conducted to assess the cellular functionality of pUVEC when seeded on the dECM discs and in 2D controls (Figure 5C). Due to the lack of cell adhesion and the cell death experienced during the initial days, the consecutive frequent medium changes may have affected NO concentration determination. However, once the culture was established, a gradual and significant increase in NO concentration was observed in the culture medium of dECM discs compared to the 2D controls at days 6 and 7 of culture. The progressive formation of microvascular structures in cell-seeded dECM liver discs (Figures 5B1–B10) and the gradual replacement of pUVEC by pMSC(M) in the 2D controls (as shown in Figures 5A5–A7), explain the reduction and absence of NO secretion during the final days of the culture period.

## 4 Discussion

In this study, we addressed a critical bottleneck in tissue engineering—the formation of functional vasculature—by isolating, characterizing, and assessing the vascular potential of porcine-derived MSC(M), SMCs, and ECs within a 3D liver dECM microenvironment. Our approach aimed to provide a physiologically relevant platform that could yield valuable insights into cellular self-organization and microvascular network formation, crucial for future tissue-engineering applications (Shaheen et al., 2019; Ribitsch et al., 2020; Anderson et al., 2021).

We successfully isolated porcine MSC(M), SMCs, and ECs, verifying their phenotypes by FC, immunofluorescence, and specific functional assays. The phenotypic and functional characterization confirmed that these cells exhibit key morphological and immunophenotypic characteristics consistent with previous studies of human and porcine equivalents. Specifically, isolated MSC(M) expressed mesenchymal markers (CD29, CD44, CD90) and demonstrated multipotency, differentiating effectively into adipocytes, osteoblasts, and chondrocytes (Ringe et al., 2002; Noort et al., 2012; Arrizabalaga and Nollert, 2017; Tvorogova et al., 2018). Likewise, isolated SMCs retained a contractile phenotype, confirmed by the expression of SM22α, αSMA, and Caldesmon, as well as mesenchymal markers, highlighting their suitability for vascular tissue engineering (Chamley-Campbell et al., 1979; Lesley et al., 1993; Frid et al., 1997; Schwartz, 1999; Adams et al., 2000; Hao et al., 2003; Ponta et al., 2003). ECs from porcine umbilical veins displayed characteristic endothelial markers (CD31, CD105, CD144, Tie-2) and functional capacity through DiI-Ac-LDL uptake, confirming their endothelial phenotype and functionality (Helmke et al., 2001; Burciaga-Nava et al., 2009; Chrusciel et al., 2011; Conway et al., 2013; Post et al., 2013; Annussek et al., 2014; Hätinen et al., 2019).

A central aspect of our study was evaluating the capability of these cells to repopulate a decellularized liver matrix and form organized vascular structures. In this context, xenogeneic rat-derived dECM liver discs were used as a proof-of-concept platform to evaluate porcine cell behavior in a controlled static *in vitro* environment. Given our aim to eventually transition to a dynamic culture in perfusion bioreactors, the use of smaller rat whole-organ liver scaffolds will allow us to reduce the number of porcine cells needed, making the system more manageable and cost-effective for initial *in vitro* optimization. This strategy will facilitate subsequent scaling toward porcine liver scaffolds under perfused conditions. Moreover, our results highlighted significant differences between cells cultured in a conventional 2D environment versus those in a 3D liver dECM scaffold. While cells in 2D culture failed to maintain spatial organization and demonstrated displacement due to differential proliferation rates, the 3D liver dECM facilitated the formation of robust microvascular-like structures. The scaffold appeared to direct cell migration and organization effectively, as evidenced by ECs forming microvessels surrounded by MSC-derived pericytes and sparsely distributed SMCs. As demonstrated by our study and supported by previous research, this spatial arrangement closely resembles natural microvascular architecture, indicating that dECM provides essential structural and biochemical cues for microvascular formation (Cleaver and Melton, 2003; Bahramsoltani et al., 2009; Baptista et al., 2011; Mazza et al., 2015; Sanz-Nogués and O’Brien, 2016; Verstegen et al., 2017).

Functionally, the increased secretion of NO by ECs seeded on the dECM discs, particularly during later culture periods, suggests maintained endothelial viability and functionality. Given NO’s critical role in vascular homeostasis and angiogenesis, the elevated NO production underscores the suitability of the 3D dECM scaffold in promoting a functionally relevant endothelial phenotype (Tousoulis et al., 2011). Conversely, the decline in NO secretion in the 2D controls further highlights the inadequacy of conventional culture methods to sustain EC functionality in multi-cellular co-cultures (Morbidelli et al., 2005; Baptista et al., 2016; Vyas et al., 2018; Sánchez-Romero et al., 2019; Takeishi et al., 2020).

Our findings emphasize the superiority of the dECM-based 3D culture system for supporting physiologically relevant cellular interactions, crucial for forming stable and functional microvascular networks. This approach represents a significant advancement over traditional and often used 2D methods and offers a robust platform for future investigations into vascular biology, angiogenesis, and potential drug-testing applications. Ultimately, this model could accelerate progress toward functional organ bioengineering, addressing critical challenges associated with organ transplantation and regenerative medicine.

## Data Availability

The original contributions presented in the study are included in the article/supplementary material, further inquiries can be directed to the corresponding author.

## Glossary

AF488: Alexa Fluor 488
ALP: Alkaline Phosphatase
DiI-Ac-LDL: 1′-dioctadecyl-3,3,3′,3′-tetramethyl-indocarbocyanine perchlorate Acetylated Low-Density Lipoprotein
DAPI: 4′,6-diamidino-2-phenylindole;
dECM: decellularized Extracellular Matrix
DMEM: Dulbecco’s Modified Eagle’s Medium
EC: Endothelial Cells
ECM: Extracellular Matrix
EDTA: Ethylenediaminetetraacetic acid
EGF: Epithelial Growth Factor
FBS: Fetal Bovine Serum
FC: Flow Cytometry
FGF-2: Fibroblast Growth Factor-2
FITC: Fluorescein isothiocyanate
GFP: Green Fluorescent Protein
HA: Hepatic Artery
HBSS: Hank’s Balanced Salt Solution
IGF-1: Insulin-like Growth Factor
MNC: Mononuclear Cells
MSC: Mesenchymal Stromal Cells
NO: Nitric Oxide
OCT: Optimal Cutting Temperature Compound
PBS: Phosphate Buffered Saline
PE: Phycoerythrin
PerCP-Cy5.5: Peridinin Chlorophyll Protein Cyanine 5.5
P/S: Penicillin/Streptomycin
PFA: Paraformaldehyde
pASMC: Porcine Aortic Smooth Muscle Cells
pMSC(M): Porcine Bone Marrow derived-Mesenchymal Stromal Cells
pEGM: Porcine Endothelial Growth Medium
pUVEC: Porcine Umbilical Vein Endothelial Cells
PV: Portal Vein
RM: Regenerative Medicine
RT: Room Temperature
αSMA: alpha Smooth Muscle Actin
SM22α: Smooth Muscle 22 alpha
SM-MHC: Smooth Muscle Myosin Heavy Chain
SMC: Smooth Muscle Cells
TBST: Tris Buffered Saline-Tween 20
TE: Tissue Engineering
VEGF: Vascular Endothelial Growth Factor
